# Effect of bortezomib on human neuroblastoma: analysis of molecular mechanisms involved in cytotoxicity

**DOI:** 10.1186/1476-4598-7-50

**Published:** 2008-06-05

**Authors:** Valérie Combaret, Sandrine Boyault, Isabelle Iacono, Stéphanie Brejon, Raphaël Rousseau, Alain Puisieux

**Affiliations:** 1 Laboratoire de Recherche Translationnelle, Centre Léon Bérard, Lyon, France; 2 Institut d'Hématologie-Oncologie Pédiatrique, Lyon, F-69008, France; 3 Faculté de Médecine Lyon-Nord, Université Claude Bernard, Lyon 1, Lyon, France; 4 Inserm, U590, Lyon, France; 5 ISPB, Université de Lyon, Lyon 1, Lyon, France

## Abstract

**Background:**

Bortezomib, a specific and selective inhibitor of the 26S proteasome with antitumor activity against a wide range of malignancies, has been approved for the treatment of relapsed or refractory multiple myeloma and other cancers. Recently, bortezomib has been identified as an effective inhibitor of neuroblastoma cell growth and angiogenesis.

**Results:**

In the present study, we demonstrate that some neuroblastoma cell lines are actually resistant to bortezomib. We have sought to characterize the main pathway by which proteasome inhibition leads to apoptosis, and to define the mechanism responsible for resistance to bortezomib in neuroblastoma cells. Our results show that SB202190, an inhibitor of mitogen-activated protein kinase (MAPK) p38, enhances the ability of bortezomib to induce apoptosis by preventing the phosphorylation of the heat shock protein (HSP) 27.

**Conclusion:**

This study opens the way to further clinical investigations and suggests a potential benefit of using a combination of bortezomib with an inhibitor of p38 MAPK for the treatment of neuroblastoma relapse.

## Background

Neuroblastoma (NB) accounts for 8% to 10% of childhood cancers [1]. The two main prognostic factors are age and stage [2,3]. Localized NB and those arising in infants have a 90% survival rate, except in cases of *MYCN *amplification where survival is below 30% [3-5]. Approximately 50% of all NB occurring in children older than 1 year are metastatic at diagnosis. NB is considered chemosensitive. Chemotherapy is indicated in localized NB for patients with large primary tumors in whom tumor chemoreduction allows safer surgical excision [6,7], as well as in metastatic NB to achieve complete remission of metastases. The most effective drugs are alkylating agents, platinum compounds, anthracyclines and epipodophyllotoxins [8]. High-dose chemotherapy followed by hematopoietic stem cell transplantation and maintenance therapy with retinoic acid improve survival by 35% in children with metastatic NB [9,10], but the 5-year event-free survival rate remains below 50%. Therefore, novel therapeutic approaches are needed.

The multicatalytic ubiquitin-proteasome pathway is responsible for the degradation of eukaryotic cellular proteins [11-14]. This adenosine 5'-triphosphate-dependent process is vital for normal cell cycling, function and survival, making proteasome inhibition a novel therapeutic strategy in cancer. The dipeptidyl boronic acid bortezomib (PS-341, Velcade^® ^Janssen Cilag, Issy-les Moulineaux, France) is a specific and selective inhibitor of the 26S proteasome [14,15]. Studies have established its antitumor activity against a wide range of malignancies, including myeloma, prostate cancer, breast cancer, colon cancer, and lung cancer [14,16]. Recently, bortezomib became the first proteasome inhibitor approved by the U.S. Food and Drug Administration for the treatment of relapsed or refractory multiple myeloma. Ongoing clinical trials of bortezomib for prostate and lung cancers have yielded promising results [17]. Recently, the effects of bortezomib on human neuroblastoma cells have been studied both *in vitro *and in nude mice [18,19]. Apoptosis, as well as cell cycle and angiogenesis inhibitions have been observed, but the molecular mechanisms by which bortezomib induces cytotoxicity in neuroblastoma have not been analyzed. The aim of our study was to characterize the main pathway by which proteasome inhibition leads to apoptosis and to define the mechanisms responsible for resistance to bortezomib in several neuroblastoma cells.

## Results

### Effect of bortezomib on the proliferation of neuroblastoma cell lines

**Figure 1:**
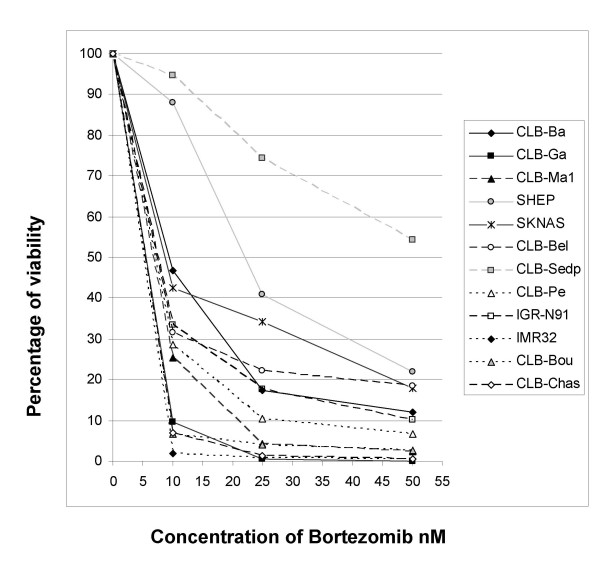
**Differential sensitivity of neuroblastoma cell lines to bortezomib**. Cell survival in the presence of increasing concentrations of bortezomib was assessed by the Uptiblue assay at 72 h of treatment.

We first investigated the effect of bortezomib on cell viability *in vitro *in 12 neuroblastoma cell lines using the Uptiblue assay. The cell lines were incubated with various concentrations of bortezomib (0 to 50 nM) for 72 hours. Results showed a dose-dependent cytotoxitic activity (figure 1). However, response to bortezomib varied significantly with the neuroblastoma cell lines tested. IMR32, IGRN91, CLB-Ga, CLB-Bou, CLB-Chas, CLB-Ma1, CLB-Pe, SKNAS, CLB-Ba and CLB-Bel cell lines displayed a half maximal inhibitory concentration (IC50) of <10 nM and were considered sensitive to bortezomib since this concentration level has been defined as clinically achievable [20] whereas CLB-Sedp and SHEP displayed higher IC50 values (> 25 nM) indicative of strong resistance to bortezomib.

### Activation of apoptotic pathways by bortezomib

**Figure 2:**
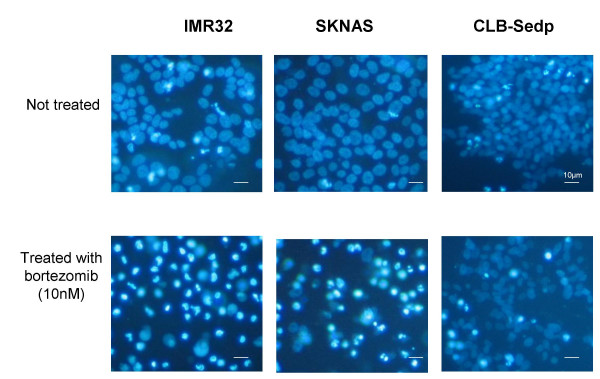
**Bortezomib induces apoptosis in neuroblastoma cells**. Sensitive (IMR32 and SKNAS) and resistant (CLB-sedp) neuroblastoma cell lines were treated with or without 10 nM bortezomib for 72 h. Cells were stained with HOECHST 33258 (2 μl/ml). Apoptotic cells were identified by condensation and fragmentation of nuclei using a Zeiss Axiovert inverted light microscope.

**Figure 3:**
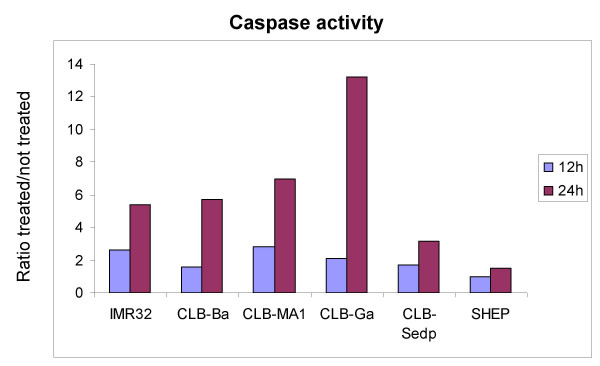
**Caspase activation**. Analyses were performed using the caspase-Glo^® ^3/7 assay. Neuroblastoma cell lines were incubated with or without bortezomib for 12 h or 24 h in four replicate wells. Results were expressed as the ratio of the mean relative light units obtained with treatment to the mean relative light units obtained without treatment.

HOECHST 33258 staining revealed classic apoptotic hallmarks such as chromatin condensation in bortezomib-sensitive neuroblastoma cell lines, as illustrated in figure 2. The percentage of apoptotic cells observed after 72 H treatment of neuroblastoma cell lines with 10 nM bortezomib was higher in sensitive than in resistant cell lines (91%, 56.5%, 37.7% and 21% for IMR32, SKNAS, CLB-Sedp and SHEP cell lines, respectively, vs. 23%, 20.7%, 19% and 17%, respectively, in the absence of treatment). When the intrinsic or mitochondrial-based cell death pathway is engaged, there is release of proapoptotic factors such as cytochrome *c *and SMAC/DIABLO [21] from the mitochondria with subsequent activation of caspase 9 and other caspases such as caspases 3 and 8 [22-24]. Release of cytochrome *c *from the mitochondria to the cytoplasm was detected in bortezomib-sensitive neuroblastoma cell lines whereas no significant variation was observed in resistant cell lines (data not shown). Similarly, a higher activation of caspase 3/7 under bortezomib treatment was found preferentially in sensitive neuroblastoma cell lines (Figure 3).

### Analysis of *p53 *mutational status

We searched whether *p53 *mutational status could explain the difference in responses to bortezomib. The sequencing of all *p53 *exons with their intron boundaries was performed in the panel of 12 neuroblastoma cell lines. Mutations were observed in CLB-Pe, CLBMa1 and SKNAS. For SKNAS, we confirmed the truncating mutation observed by others [25,26] with deletion of exons 10 and 11. Misense mutations were observed in other cell lines corresponding to aminoacid substitutions at codon 176 (C176F) in CLB-Pe and codon 235 (N235S) in CLB-Ma1.

### Induction of proapoptotic and antiapoptotic factors under bortezomib treatment

**Figure 4:**
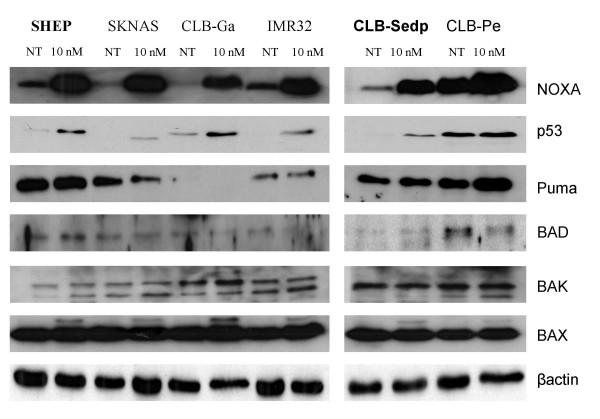
**Changes in the expression of proapoptotic factors after bortezomib treatment**. Neuroblastoma cell lines were examined either before (NT, not treated) or after 24 h of exposure to bortezomib (10 nM) and a series of Western blots (see methods) using specific antibodies was performed to detect relative levels of proteins. The 2 resistant neuroblastoma cell lines are mentioned in bold type.

**Figure 5:**
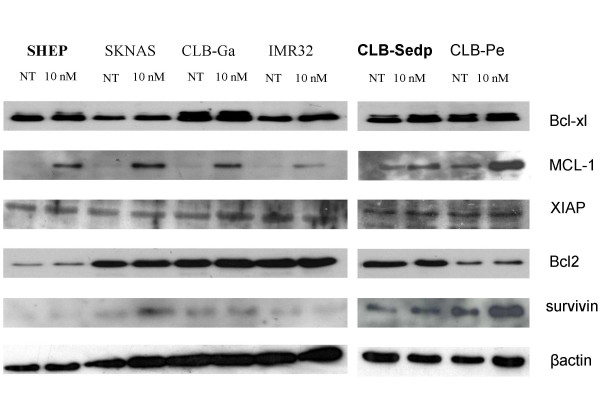
**Changes in the expression of antiapoptotic factors after bortezomib treatment**. Neuroblastoma cell lines were examined either before (NT) or after 24 h of exposure to bortezomib (10 nM) and a series of Western blots (see Methods) using specific antibodies was performed to detect relative levels of proteins. The two resistant neuroblastoma cell lines are mentioned in bold type.

Given that differences in the response to bortezomib could not be explained by *p53 *mutational status, we searched whether there was a difference among regulators of apoptosis. Among the pro-apoptotic factors studied (p53, Noxa, PUMA, Bad, Bax, and Bak), only NOXA was consistently induced in 6 bortezomib-treated neuroblastoma cell lines. An accumulation of the p53 protein was observed in all cell lines except CLB-Pe which displayed a mutated *p53*. For SKNAS, an abnormal p53 protein was observed. These data were consistent with the results obtained in the analysis of *p53 *mutational status. For other apoptotic proteins (PUMA, Bad, Bax and Bak), no changes in expression levels were observed (figure 4). The antiapoptotic factors analyzed were Bcl2, Bcl-xl, Mcl-1, XIAP and survivin. Mcl-1 was upregulated after bortezomib exposure in all neuroblastoma cell lines whereas no change was identified in Bcl2, XIAP, survivin and Bcl-xl expression after bortezomib treatment (figure 5).

### Induction of heat shock proteins under bortezomib treatment

**Figure 6:**
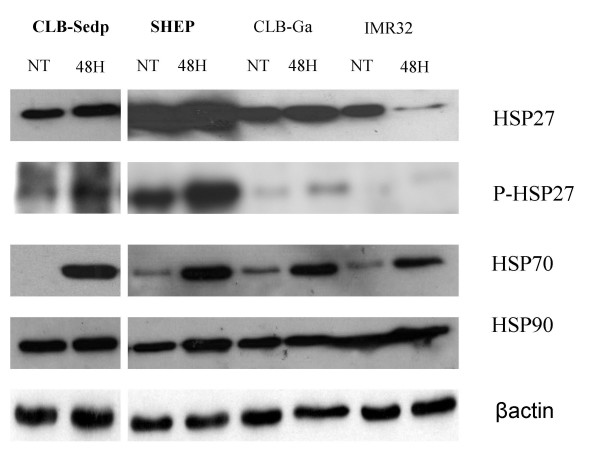
**Expression of heat shock proteins**. Neuroblastoma cell lines were examined either before (NT) or after 48 h of exposure to bortezomib (10 nM). Western blots using specific antibodies (see Methods) were performed to determine the expression of HSP27, P-HSP27, HSP70, HSP90. The two resistant neuroblastoma cell lines are mentioned in bold type.

No obvious difference was detected between bortezomib-sensitive and -resistant neuroblastoma cell lines during the analysis of apoptotic and antiapoptotic factors. We examined whether other proteins could be involved in the resistance to bortezomib. Microarray comparisons of the transcriptional profile of lymphoma cells resistant or sensitive to bortezomib have shown that the overexpression of heat shock proteins (HSP) is associated with bortezomib resistance in this cell type [27,28]. We studied the expression of HSP27, phosphorylated HSP27, HSP70 and HSP90 in the bortezomib-sensitive and -resistant NB cell lines. HSP27 expression was detected in both sensitive and resistant cells lines before and after bortezomib treatment. A high pre-treatment expression and a post-treatment induction of phosphorylated HSP27 (P-HSP27) was observed in the resistant cell lines SHEP and CLB-Sedp, whereas only moderate expression was found in sensitive cell lines (figure 6). HSP70 was induced in all neuroblastoma cell lines treated with bortezomib. No change in HSP90 expression could be observed in any neuroblastoma cell line after bortezomib treatment (figure 6)

### Mitogen-activated protein kinase p38 inhibition enhances bortezomib-induced cytotoxicity against neuroblastoma cell lines by inhibition of HSP27 phosphorylation

**Figure 7:**
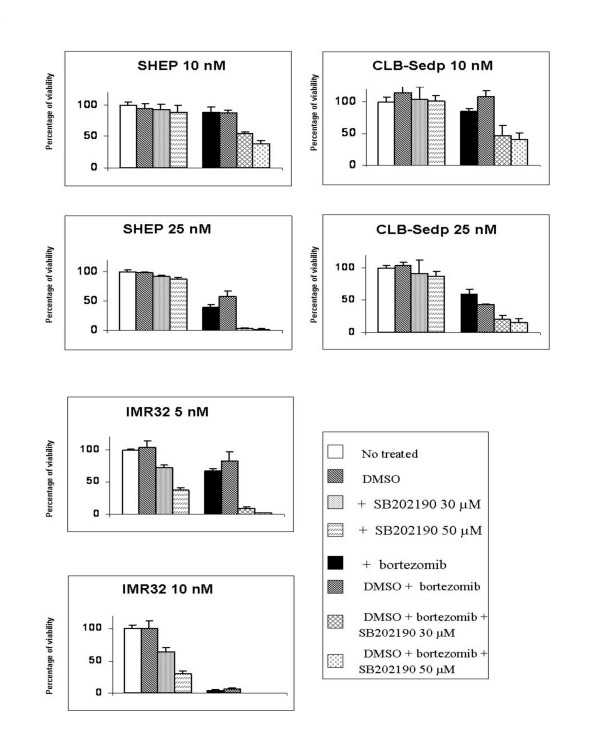
**Inhibition of p38 MAPK increases cell death in bortezomib-resistant neuroblastoma cell lines**. The neuroblastoma cell lines SHEP, CLB-Sedp and IMR32 were cultured in the absence or presence of SB202190 (p38 MAPK inhibitor) for 30 min prior to culture with different concentrations of bortezomib for 72 h. Cell viability was assessed using the Uptiblue assay.

**Figure 8:**
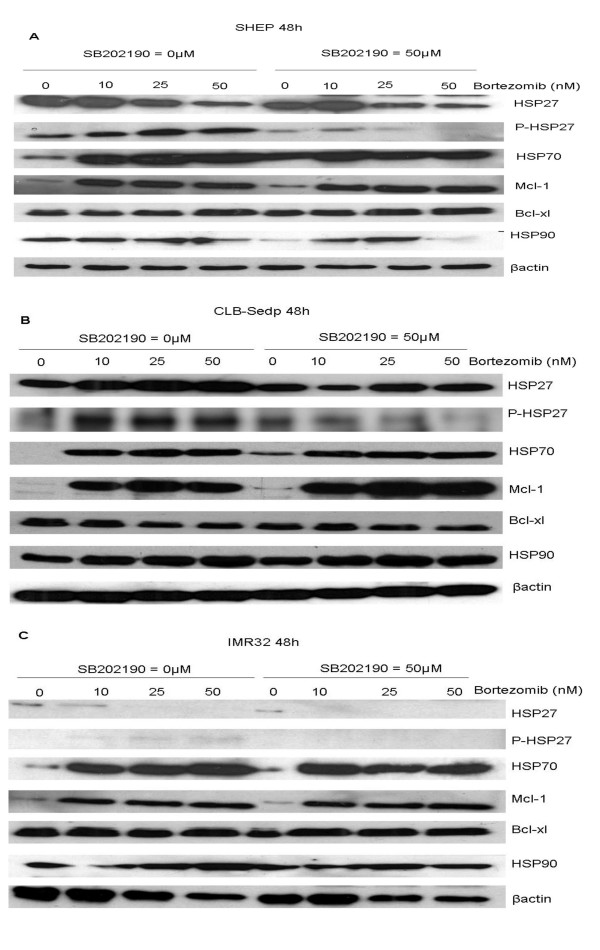
**Down regulation of HSP27 phosphorylation by inhibition of p38 MAPK**. The neuroblastoma cell lines SHEP, CLB-Sedp and IMR32 were treated with SB202190 (50 μM) for 30 min prior to culture with different concentrations of bortezomib for 48 h. Expressions of HSP27, P-HSP27, HSP70, HSP90, Mcl1 and Bcl-xl were assessed by Western blot using specific antibodies.

As reported above, bortezomib up regulates P-HSP27 expression in resistant neuroblastoma cell lines. Previously, Hideshima *et al*. [29] have shown that MAPK p38 inhibition enhances the ability of bortezomib to induce apoptosis in multiple myeloma cells. Since p38 MAPK phosphorylates HSP27, thereby enhancing its activity, we tested whether SB202190, a p38 MAPK inhibitor, can increase bortezomib-induced cytotoxicity in neuroblastoma cell lines. The cell lines were first incubated for 30 min with various concentrations of SB202190 then treated with different concentrations of bortezomib. The combination of drugs increased cytotoxicity in a dose-dependent manner (figure 7). For SHEP neuroblastoma cells, the cytotoxic effect was initiated when SB202190 was used in association with 10 nM bortezomib and increased when SB202190 was used in association with 25 nM bortezomib. As shown on figure 8A, this cytotoxic effect was correlated to the inhibition of HSP27 phosphorylation since a decrease of P-HSP27 expression was observed from 25 nM bortezomib when cells were preincubated with SB202190. The same observation could be made for CLB-Sedp although in this cell line the association of SB202190 with bortezomib resulted in lower cytotoxicity (Figure 7). Indeed P-HSP27 protein is still slightly expressed when the two drugs are used in association (Figure 8B). Consequently, resistance to bortezomib appears to depend on the phosphorylation of Hsp27. Although a low level of P-HSP27 expression was detected in the IMR32 cell line, we observed that SB202190 also reduced the phosphorylation of HSP27 (Figure 8C) and improved the cytotoxic effect of bortezomib. Indeed, 5 nM bortezomib combined with 50 μM SB202190 were as efficient as 10 nM bortezomib (Figure 7).

## Discussion

Bortezomib is a proteasome inhibitor showing antitumor activity in a wide range of malignancies. Despite abundant evidence of the therapeutic potential of this drug, the relevant signaling pathways leading to apoptosis in cancer cells are not clear. In this study, we demonstrate that bortezomib induces the apoptosis of neuroblastoma cells by activation of caspases. However, the response to the drug varies depending on the neuroblastoma cell lines tested. Because concentration levels below 10 nM can be observed in bortezomib-treated patients, as previously reported by Rajkumar *et al *[20] and Boccadoro *et al *[30], 10 of the 12 neuroblastoma cell lines analyzed were indeed identified as sensitive (IC50 <10 nM) whereas 2 were resistant (IC50 >25 nM) to bortezomib. The response to the drug appears to be independent of the p53 mutational status. Indeed, the three neuroblastoma cell lines for which a p53 mutation has been identified by sequencing (CLB-Pe, CLB-Ma1 and SKNAS) and the IGR-N91 cell line for which a p53 mutation has been identified by northern blot and western blot analyses [26,31] remained sensitive to bortezomib. These data confirm the results obtained by others in a variety of cancers [18,19,32-34].

We addressed the question of which molecular mechanisms were responsible for the induction of apoptosis by bortezomib in neuroblastoma cell lines. We searched whether members of the BCL2 family or other known regulators of apoptosis were involved. The analysis of proapoptotic factors such as p53, NOXA, Bad, PUMA, Bax or Bak, and of antiapoptotic factors such as Bcl2, Bcl-xl, Mcl-1, XIAP and survivin failed to explain the differences between NB cell lines. The accumulation of p53 and induction of NOXA and Mcl-1 were not associated with any major difference in response to bortezomib. Different teams [33,34] have shown that bortezomib induces the proapoptotic BH3-only family member NOXA in a p53 independent fashion and have identified NOXA as a key element in the triggering of a caspase cascade culminating in apoptosis in melanoma and myeloma cells. They have also demonstrated the induction of Mcl-1 and survivin, two antiapoptotic proteins known to decrease cell sensitivity to bortezomib [35,36]. The overexpression of Mcl-1 reduces bortezomib-induced apoptosis by interacting with NOXA and exerts its anti-apoptotic effect by inhibiting caspase-9 activity. Our observation of an induction of NOXA in *p53*-mutated neuroblastoma cell lines confirmed the previous demonstration that induction of NOXA after bortezomib exposure is p53-independent. However, the resistant neuroblastoma cell lines tested in our study showed reduced apoptosis in spite of high NOXA induction, thus indicating that NOXA is not sufficient to trigger apoptosis. Induction of the antiapoptotic protein Mcl-1 cannot be the reason for this low apoptosis reported in resistant neuroblastoma cell lines since the protein is equally expressed in sensitive and resistant cells after bortezomib exposure.

Abnormal HSP expression has been shown to be implicated in both chemotherapeutic resistance and carcinogenesis [37-39] and increased HSP activity appears to result in aggressively growing, therapy-resistant tumors [40-42]. Mitsiades *et al*. [43] have demonstrated that bortezomib significantly upregulates antiapoptotic HSP in multiple myeloma cells in a time-dependent manner. Moreover, a number of constitutively activated growth signaling pathways has been shown to play crucial roles in regulating cell growth, metabolic responses, cell proliferation, migration and apoptosis. Constitutive activation of protein kinases, mainly by phosphorylation, contributes to malignant phenotypes in a number of human cancers. Although the p38 MAPK pathway has been associated with the induction of apoptosis in response to various cellular stresses such as treatment with anticancer drugs [44], p38MAPK activation is also central to antiapoptotic and growth-promoting effects. For instance, Nemoto *et al*. [45] have suggested that distinct members of the p38 MAPK family have different functions in apoptosis. p38α has been identified as a mediator and p38β as an inhibitor of apoptosis. Other teams have described the antiapoptotic role of p38 MAPK in a number of cell types [46,47]. p38 MAPK is known to phosphorylate HSP27, *via *either MAPK-activated protein kinase 2 (MAPKAPK2) and/or p38 MAPK-regulated/activated protein kinase (PRAK) [48,49]. Our study shows an expression of phosphorylated-HSP27 before bortezomib treatment and its induction after treatment in resistant cell lines. Combined exposure to bortezomib and to the p38 MAPK inhibitor SB202190 leads to enhanced apoptosis in neuroblastoma cell lines defined as resistant to bortezomib but also improves the cytotoxic effect observed in sensitive cell lines. p38 MAPK inhibition prevents the phosphorylation of the antiapoptotic protein HSP27 but does not influence its induction, as mentioned by others [29]. Navas *et al*. [50] have reported that p38 MAPK inhibition enhances the cytotoxicity of bortezomib for multiple myeloma cells both by inhibiting the transient expression and phosphorylation of HSP27 and by down regulating Bclxl and Mcl-1 expression. Our data confirm the importance of HSP27 phosphorylation for resistance to bortezomib. However, as no modification in Bclx and Mcl-1 expression was observed under treatment with p38 MAPK inhibitor (Figure 8), we could not confirm that these two proteins are involved in resistance to bortezomib. A study comparing bortezomib-resistant and bortezomib-sensitive large B cell lymphoma cell has identified the molecular markers correlated with sensitivity or resistance to bortezomib treatment. The overexpression of activating transcription factor 3 (ATF3), ATF4, ATF5, c-Jun, JunD and caspase-3 is associated with bortezomib-induced apoptosis whereas the overexpression of heat shock proteins HSP27, HSP70, HSP90 and T-cell factor 4 is associated with bortezomib resistance [29]. Neither HSP70 or HSP90 influenced cell resistance to bortezomib in our neuroblastoma model. Our study shows that P-HSP27 is a key element that promotes cell survival by inhibiting apoptosis, and confirms the results obtained by other teams [51-53]. Different targets of p38 MAPK have already been identified and we cannot exclude that other elements could be involved in the apoptosis observed after combined SB202190-bortezomib treatment. Indeed, Nemoto *et al*. [45] have reported that SB202190 is able to potentiate the apoptosis induced by Fas (APO1) ligation or UV irradiation *via *the stimulation of CPP32-like caspases. Furthermore, Shi *et al*. [54] have shown that the inhibition of p38 MAPK results in the suppression of anti apoptotic MAPK phosphatase-1 induction.

## Conclusion

We have shown that most neuroblastoma cell lines are sensitive to bortezomib treatment. The association of bortezomib with a p38 MAPK inhibitor can induce cell death in resistant cells. Various p38 MAPK inhibitors have been developed [55]. We suggest that combinations of bortezomib with one of the p38 MAPK inhibitors currently available could be used in relapsing neuroblastoma patients to increase treatment response.

## Methods

### Reagents

Bortezomib (PS-341, Velcade^®^) was provided by Janssen Cilag (Issy-les Moulineaux, France) as a lyophilized white cake in 10-mL glass vials. Each sterile single-use vial contained 3.5 mg bortezomib and 35 mg mannitol. Vials were stored at +25°C. For *in vitro *experiments, the content of each vial was dissolved in 3.5 mL of NaCl buffer (0.9%) to a final concentration of 2.6 mM bortezomib. The reconstituted drug was stored at -80°C then thawed and diluted in culture medium immediately before use. SB202190, a specific inhibitor of the mitogen-activated protein kinases (MAPK) p38α and p38β, was purchased from Sigma Aldrich (Saint Quentin Fallavier, France). Primary antibodies to Bcl2, BclXL, p53 (Dako, Trappes, France), HSP27, P-HSP27, HSP70, HSP90, p38 MAPK (Cell signaling Technology, Beverly, MA, USA), Puma (ABcam, Paris, France), NOXA (Calbiochem, VWR International, Fontenay-sous-bois, France), Bax, Bak, survivin (SantaCruz Biotechnology, SantaCruz, CA, USA), Bad (Serotec, Cergy Saint-Christophe, France), XIAP (Stressgen Biotechnologies, Victoria, Canada), cytochrome c and Mcl-1 (BD Biosciences, Pont de Claix, France), β actin (Sigma Aldrich) were used at dilutions recommended by their respective manufacturers.

### Cell lines and cell culture

The neuroblastoma cell lines CLB-Ga, CLB-Bou, CLB-Chas, CLB-Ma1, CLB-Pe, CLB-Ba, CLB-Bel and CLB-Sedp were established in our laboratory as previously described [56]. IMR32 and SKNAS were obtained from the American Type Culture Collection (ATCC, Manassas, VA, USA), and SHEP and IGR-N91 were kindly provided by M. Schwab and J. Benard, respectively. All cell lines were grown in RPMI 1640 (Gibco-Invitrogen, Cergy-Pontoise, France) supplemented with 10% heat-inactivated fetal bovine serum (Cambrex-Biowhittaker, Emerainville, France) and 200 IU/ml penicillin, 200 μg/ml streptomycin and 2 mM L-glutamine (all reagents from Gibco-Invitrogen) at +37°C, under 5% CO_2_. Cells were treated with various concentrations of bortezomib and/or SB202190 during different time intervals.

### Cell Viability Assay

Cell viability at 72 h was assessed using the Uptiblue assay (Interchim, Montluçon, France) according to the manufacturer's instructions. Cells were seeded in 96-well plates (2 × 10^4 ^cells/well). Five replicate wells were used for each experimental condition. Different concentrations of bortezomib were tested. Experiments were performed in triplicate for each cell line.

### *p53 *mutation analysis

Total cellular DNA was extracted from frozen cell pellets using phenol chloroform extraction. Exons 1 to 11 of the *p53 *gene were amplified by PCR. Detailed sequences of the primers and PCR protocols are available upon request. The PCR products were purified with a Millipore PCR purification kit (Millipore, Saint Quentin en Yvelines, France) and sequenced on a 3700 sequencer (Applied Biosystems, Foster City, CA) using the BigDye Terminator sequencing kit (Applied Biosystems). Sequences were analyzed using SeqScape software (Applied Biosystems).

### Visualization of chromatin condensation (HOECHST 33258 staining)

Neuroblastoma cells were treated with bortezomib (10 nM) for 72 h. Eighteen hours before visualization, HOECHST 33258 (Sigma) was added to the culture medium. Apoptotic cells were identified by condensation and fragmentation of nuclei using a Zeiss Axiovert inverted light microscope (Carl Zeiss SAS, Le Pecq, France). Images were captured using a Power ShotG5 digital camera (Canon, Courbevoie, France).

### Western Blot analysis

Cell pellets were lysed 30 min on ice in cold lysis buffer (50 mM Tris-Hcl [ph = 7.4], 0.25 mM NaCl, 1 mM CaCl2, 50 mM NaF, 0.2% NP40) containing a protease inhibitor cocktail (Roche, Meylan, France). Cell lysates were then cleared by centrifugation. For the analysis of cytochrome c release, cell pellets were resuspended in digitonin extraction buffer (phosphate-buffered saline (PBS) containing 250 mM sucrose, 70 mM KCl, 1 mM phenylmethylsulfonyl fluoride, 200 μg digitonin/mL, and 1 protease inhibitor cocktail tablet per 10 mL) and incubated on ice for 5 min. Samples were then centrifuged at 1,000 *g *for 5 min; the pellet was removed and the supernatant was collected and centrifuged again at 12,000 *g *for 15 min. The supernatant (cytosolic fraction) was collected and the pellet (mitochondrial fraction) was lysed in RIPA lysis buffer (PBS containing 1% NP-40, 0.5% Na-deoxycholate, and 0.1% sodium dodecyl sulfate [SDS]) supplemented with 1 mM dithiothreitol, 1 mM phenylmethylsulfonyl fluoride, and 1 protease inhibitor cocktail tablet per 10 mL. The protein concentrations of all extracts were determined using the Bio-Rad protein assay kit (Bio-Rad, Marnes-la Coquette, France) according to the manufacturer's instructions. Total protein (25–60 μg) was separated on SDS polyacrylamide gel electrophoresis and transferred to polyvinylidene fluoride membrane. After transfer completion, the membrane was blocked by incubation in 5% dry milk in TBST (0.05% Tween 20 in TBS) and probed with the primary antibodies, then with horseradish peroxidase-conjugated secondary antibodies. Blots were visualized using the ECL system (Pierce, Rockford, IL, USA).

### Analysis of caspase activation

Caspase activation was determined using the caspase-Glo^® ^3/7 assay (Promega, Charbonnières, France) according to the manufacturer's instructions. Cells (2 × 10^4 ^cells/well) were seeded in black-walled 96-well plates (Greiner, Dutscher, Issy les Moulineaux, France) treated for 12 or 24 hours with 50 nM bortezomib and incubated 1 hour at room temperature. Luminescence was measured with the Cytofluor 4000 multiwell plate reader (Applied Biosystems)

## List of abbreviations

HSP: heat shock protein; P-HSP27: phosphorylated HSP27; IC50: half maximal inhibitory concentration; MAPK: mitogen-activated protein kinase; NB: neuroblastoma; PBS: phosphate-buffered saline; SDS: sodium dodecyl sulfate.

## Competing interests

The authors declare that they have no competing interests.

## Authors' contributions

VC conceived the study, participated in its design and drafted the manuscript. SB participated in the analysis of p53 mutational status. II and SB participated in all cell culture experiments and performed the immunoblots and caspase activity assay. RR and AP were involved in the overall design of the study and helped to draft the manuscript
